# Reducing the serine availability complements the inhibition of the glutamine metabolism to block leukemia cell growth

**DOI:** 10.18632/oncotarget.6426

**Published:** 2015-11-28

**Authors:** Florence Polet, Cyril Corbet, Adan Pinto, Laila Illan Rubio, Ruben Martherus, Vanesa Bol, Xavier Drozak, Vincent Grégoire, Olivier Riant, Olivier Feron

**Affiliations:** ^1^ Pole of Pharmacology and Therapeutics (FATH), Institut de Recherche Expérimentale et Clinique (IREC), Université catholique de Louvain, B-1200 Brussels, Belgium; ^2^ Pole of Medical Imaging, Radiotherapy and Oncology (MIRO), Institut de Recherche Expérimentale et Clinique (IREC), Université catholique de Louvain, B-1200 Brussels, Belgium; ^3^ Molecules, Solids and Reactivity (MOST), Institute of Condensed Matter and Nanosciences (IMCN), Université catholique de Louvain, B-1348 Louvain-la-Neuve, Belgium

**Keywords:** glutamine, serine, PHGDH, metabolism, leukemia

## Abstract

Leukemia cells are described as a prototype of glucose-consuming cells with a high turnover rate. The role of glutamine in fueling the tricarboxylic acid cycle of leukemia cells was however recently identified confirming its status of major anaplerotic precursor in solid tumors. Here we examined whether glutamine metabolism could represent a therapeutic target in leukemia cells and whether resistance to this strategy could arise. We found that glutamine deprivation inhibited leukemia cell growth but also led to a glucose-independent adaptation maintaining cell survival. A proteomic study revealed that glutamine withdrawal induced the upregulation of phosphoglycerate dehydrogenase (PHGDH) and phosphoserine aminotransferase (PSAT), two enzymes of the serine pathway. We further documented that both exogenous and endogenous serine were critical for leukemia cell growth and contributed to cell regrowth following glutamine deprivation. Increase in oxidative stress upon inhibition of glutamine metabolism was identified as the trigger of the upregulation of PHGDH. Finally, we showed that PHGDH silencing *in vitro* and the use of serine-free diet *in vivo* inhibited leukemia cell growth, an effect further increased when glutamine metabolism was blocked. In conclusion, this study identified serine as a key pro-survival actor that needs to be handled to sensitize leukemia cells to glutamine-targeting modalities.

## INTRODUCTION

Leukemia represents the most common cancer in children and is the sixth leading cause of death for adults [1, 2]. Although hematological malignancies comprise a collection of heterogeneous diseases, they all originate from the hematopoietic system and undergo a particularly high turnover rate. This generally accounts for a good rate of response to anti-proliferative chemotherapy. Still, poorly responding leukemia and acquisition of resistance justify the need to identify new modalities of treatment. Among the possible new therapeutic targets there is an increasing interest for the metabolism of leukemia cells that is necessary to sustain cell growth and proliferation [3].

It is known for about one century that leukemia cells, like most rapidly dividing cells, have a high rate of glucose (Glc) uptake. Glc is used to feed the glycolytic pathway up to the conversion of pyruvate to lactate, a process occurring even in the presence of oxygen [4]. Because of this observation, also called the Warburg effect, tumor cells and in particular leukemia cells have been considered to limitedly rely on mitochondrial oxidative phosphorylation. This has led to underestimate for decades the role of the tricarboxylic acid (TCA) cycle in the generation of biosynthetic intermediates. Recent studies in solid tumors have however demonstrated the importance of glutamine (Gln) to fuel the TCA cycle (anaplerosis), thereby offering a continuous source of precursors for lipids, aminoacids and nucleotides [5–9].

These data suggest that the blockade of the glutamine metabolism through inhibition of either Gln transport or its conversion into glutamate [10–12], could represent a safer alternative to the blockade of glycolysis which may induce metabolic disorders in healthy organs. This is further supported by the lack of alterations in blood cell count parameters in mice treated with a glutaminase inhibitor [13]. Tumor metabolic plasticity however may facilitate the adaptation of cancer cells to therapies aiming to inhibit Gln metabolism. A study by Willems *et al.* (2013) has for instance reported that the inhibition of glutaminase activity upon the administration of asparaginase (L-ase) led to an upregulation of glutamine synthase (GS) expression in leukemia cells [14], thereby reducing the therapeutic potential of this strategy. Also, Zhang and colleagues (2014) recently reported that the activity of asparagine synthetase (ASNS) was necessary to confer resistance to Gln starvation in neuroblastoma [15]. It is however unclear whether the reported L-ase anticancer effects are due to a reduction in the Asn or Gln pools, or both [16, 17].

Here, after evaluating the Glc *vs.* Gln dependence of various leukemia cells, we used two-dimensional difference gel electrophoresis (2D-DIGE) to identify differentially expressed proteins that could participate in the survival of leukemia cells following Gln deprivation. This led us to identify the upregulation of two enzymes of the serine pathway as a response to Gln starvation, namely PHGDH (phosphoglycerate dehydrogenase) and PSAT (phosphoserine aminotransferase). We found that both the exogenous serine and intracellular serine synthesis were critical for leukemia cell growth and contributed to the resistance to the pharmacological inhibition of the glutamine metabolism. Finally, we documented both *in vitro* and *in vivo* that inhibitors of the glutamine metabolism gained in being associated with PHGDH silencing or serine-free diet.

## RESULTS

### Glucose but also glutamine withdrawal inhibits leukemia cell growth

**Figure 1:**
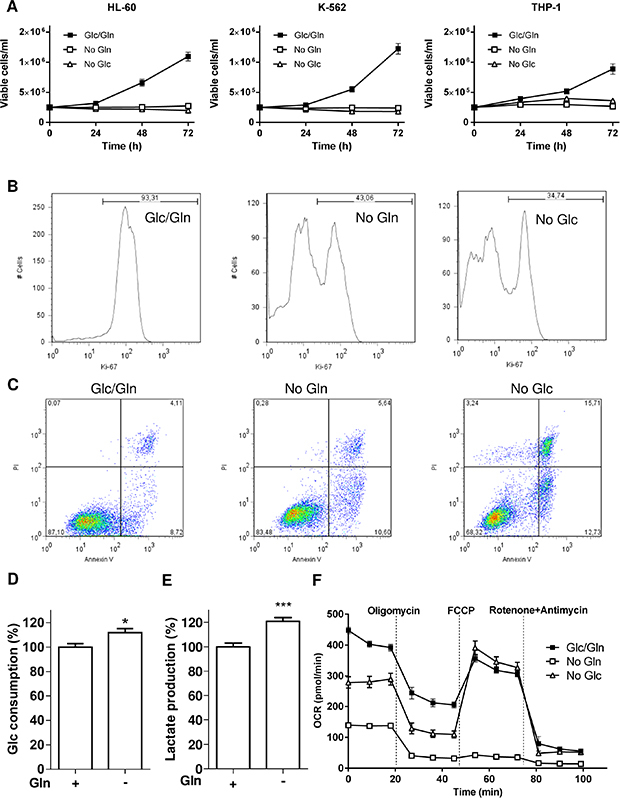
Glc and Gln are both required to support leukemia cell growth Leukemia cells (HL-60, K-562 and THP-1) were exposed to medium containing either glucose or glutamine or both. (**A**) Time course of cell growth (*n* = 3). Representative flow cytometry (**B**) histograms of Ki-67 labelled-HL-60 cells and (**C**) dot plots for Annexin V/PI labelling of HL-60 cells treated as indicated for 48 hours; these experiments were repeated twice with similar results. Bar graphs represent (**D**) the glucose consumption and (**E**) the lactate release (%, normalized per cell number) in HL-60 cells deprived or not of Gln for 48 hours (*n* = 3). (**F**) Representative graphs of OCR outputs from the Seahorse analyzer of HL-60 cells treated as indicated (*n* = 6).

To compare the role of glucose (Glc) and glutamine (Gln) for cell growth, we first cultured three different leukemia cell lines (HL-60, K-562 and THP-1) in normal medium deprived or not of Glc or Gln. We found that each cell line was similarly dependent on Glc and Gln to support cell growth (Figure 1A). Ki-67 labelling confirmed that leukemia cell proliferation was inhibited in the absence of either Glc or Gln (Figure 1B). Cell cycle studies performed on HL-60, K-562 and THP-1 cells also indicated a dramatic reduction in the proportion of leukemia cells in S-phase when either Glc or Gln was withdrawn from the culture medium (not shown). The extent of cell death as determined by differential Annexin V/PI labelling was also evaluated in the absence of either energy fuel (Figure 1C). For this parameter, the lack of Glc was significantly more detrimental than Gln starvation (Annexin V^+^/PI^+^ cell quadrant: 5.2% *vs.* 2.8% after 24 h and 15.7% *vs.* 5.6% after 48 h, respectively), suggesting that the deprivation in glutamine inhibited cell growth via *cytostatic* effects rather *than cytotoxicity*.

### Glc metabolism does not compensate for Gln starvation in leukemia cells

Because of this apparent resistance to cell death in the absence of Gln, we next examined whether an increase in Glc consumption could compensate for the deficit in Gln. A very limited increase in Glc metabolism was observed with a small increase in Glc consumption but also in lactate release in Gln-deprived HL-60 cells (Figure 1D and 1E), indicating that glucose was not diverted to fuel the respiration in the absence of Gln; similar results were obtained in K-562 and THP-1 (not shown). This was further supported by Seahorse-based measurements of the oxygen consumption rate (OCR) that was largely reduced in the absence of Gln (Figure 1F), the OCR difference between pre- and post-oligomycin treatment reflecting the contribution of OCR to ATP production (see Supplementary Figure 1A). Evaluation of glycolysis through the measurement of the extracellular acidification rate (ECAR) (See Supplementary Figure 1B) also revealed that glucose metabolism failed to compensate for the glutamine deprivation (Supplementary Figure 1C).

### Reduction in Gln availability inhibits leukemia cell growth but not survival

**Figure 2:**
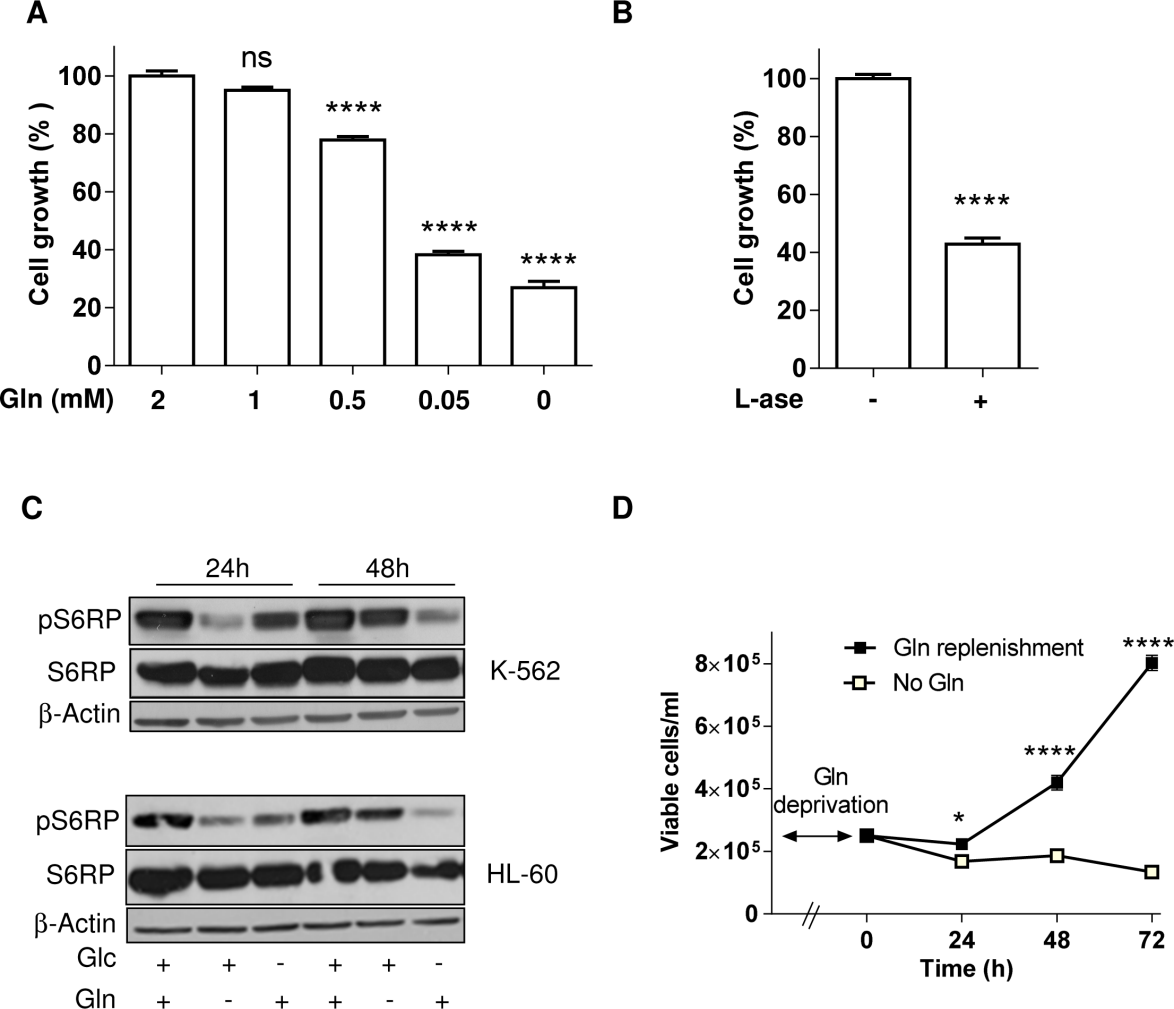
Gln deprivation inhibits leukemia cell growth but not survival Bar graphs represent the extent of HL-60 cell growth (%) for 48 hours in medium containing (**A**) the indicated concentration of Gln or (**B**) L-asparaginase (1UI/ml) (*n* = 3). (**C**) Representative immunoblots depicting the extent of phospho-S6RP in HL-60 and K-562 leukemia cell lines incubated for 24 and 48 hours in medium containing either glucose or glutamine or both; these experiments were repeated twice with similar results. (**D**) Effect of Gln replenishment on HL-60 cell growth after 72 hours Gln withdrawal (*n* = 6).

Although the need of Glc for leukemia cells to proliferate is well described, a similar strict dependence on Gln is largely unexplored. In the next series of experiments, we thus examined the effects of a reduction in Gln concentration on the proliferation and survival of leukemia cells. We found that the inhibition of cell growth was dose-dependent in the physiological range of circulating glutamine [18] (Figure 2A). We then replaced the glutamine deprivation by the use of L-asparaginase (L-ase), a treatment used in the clinics to treat ALL that was recently reported to reduce the availability of Asn but also of Gln [14]. L-ase (1UI/ml) led to the inhibition of cell growth to the same extent as observed following Gln deprivation (Figure 2B); note that an effect on the pool of extracellular Asn could be excluded since DMEM does not contain Asn. Furthermore, glutamate dosage in the extracellular medium confirmed that at the end of the incubation period with L-ase, Gln was completely degraded (2.47 mM glutamate *vs.* 0.12 mM in the presence or the absence of L-ase, respectively).

In regard to the critical role of mTOR in sensing the needs of proliferating cells in a variety of energy fuels, we then focused on the mTOR pathway. Interestingly, we observed that in K-562 and HL-60 leukemia cells, while 24 h Gln withdrawal led to an inhibition of the mTOR pathway (as probed by the downstream inhibition of the phosphorylation of S6RP), a compensatory mechanism restored (in the next 24 hours) the phospho-S6RP signal despite the maintained Gln withdrawal (Figure 2C). Glucose withdrawal, by contrast, led to a progressive non-opposed inhibition of the mTOR pathway (Figure 2C). In agreement with this apparent resistance mechanism we also documented that glutamine withdrawal stimulated autophagy (Supplementary Figure 1D) and that full medium addition could restore cell growth (Figure 2D).

### Gln withdrawal leads to the upregulation of the serine pathway

**Figure 3:**
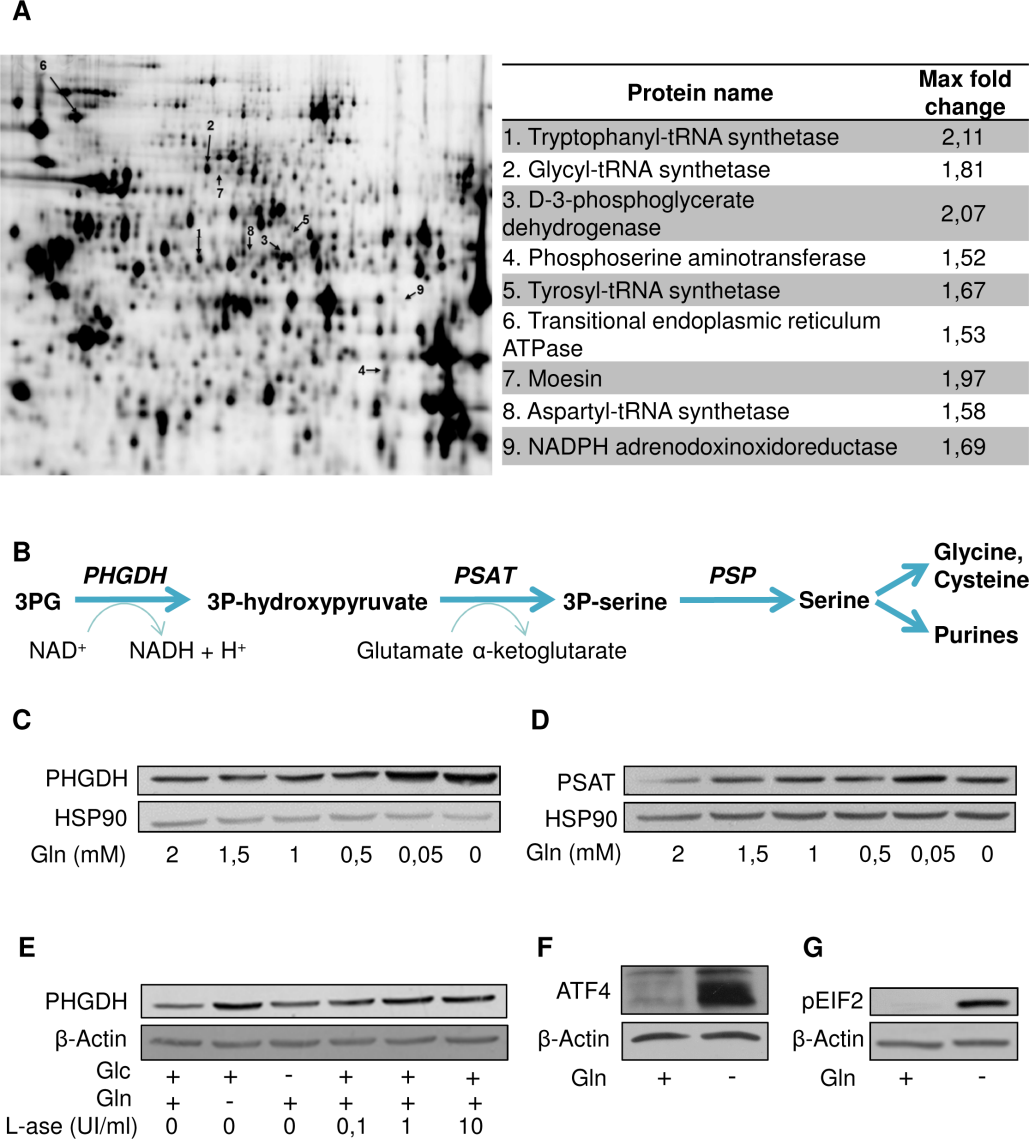
Gln deprivation promotes the serine pathway (**A**) Representative gel resulting from a 2D-DIGE experiment comparing the proteome of HL-60 cultured in the absence of Gln or Glc; proteins overexpressed in the absence of Gln indicated by an arrow (left) are listed (right) with indication of the fold-change (*P* < 0.05). (**B**) Scheme of the serine pathway from 3-phosphoglycerate (3PG). Representative immunoblots for (**C**) PHGDH and (**D**) PSAT in HL-60 leukemia cells incubated for 48 hours in medium containing the indicated decreasing concentrations of Gln. (**E**) Representative immunoblots for PHGDH in HL-60 leukemia cells exposed for 48 hours (in medium containing Glc and/or Gln) to the indicated increasing concentrations of L-asparaginase. Representative immunoblots for (**F**) ATF4 and (**G**) phospho-eIF2 after 6 hours Gln withdrawal in HL-60 leukemia cells. The immunoblot experiments were repeated 2–3 times with similar results.

The above re-growth phenomenon led us to postulate that leukemia cells may adapt and resist the inhibition of glutamine uptake. We therefore designed a 2D-DIGE experiment to compare the proteome of HL- 60 cells incubated in a medium deprived of Glc or Gln (Figure 3A). Several proteins were upregulated in the absence of Gln (Figure 3A, right panel). Among them, we identified aminoacyls (tryptophanyl-, glycyl-, tyrosyl-, aspartyl-) tRNA synthetases that are enzymes catalyzing the esterification of corresponding amino acids on tRNA. Also in response to Gln deprivation we observed the upregulation of two enzymes of the serine pathway, namely PHGDH (3-Phospholgycerate dehydrogenase) and PSAT (Phosphoglycerate aminotransferase) (Figure 3B).

We further documented in immunoblot experiments that the upregulation of PHGDH and PSAT was inversely proportional to the amount of glutamine in the medium (Figure 3C and 3D); we obtained similar results in other leukemia cell lines (Supplementary Figure 2A and 2B) and also when using L-ase (Figure 3E and Supplementary Figure 2C–2D). A strong upregulation of ATF4, a transcription factor known to directly regulate PHGDH and PSAT expression was further identified (Figure 3F) and confirmed by the detection of increased amount of phospho-EIF2, a well-known activator of ATF4 (Figure 3G). Of note, the PHGDH upregulation was not an acute effect since it was still observed several weeks after the initiation of the Gln starvation (not shown).

### Both exogenous and endogenous serine support leukemia cell growth

**Figure 4:**
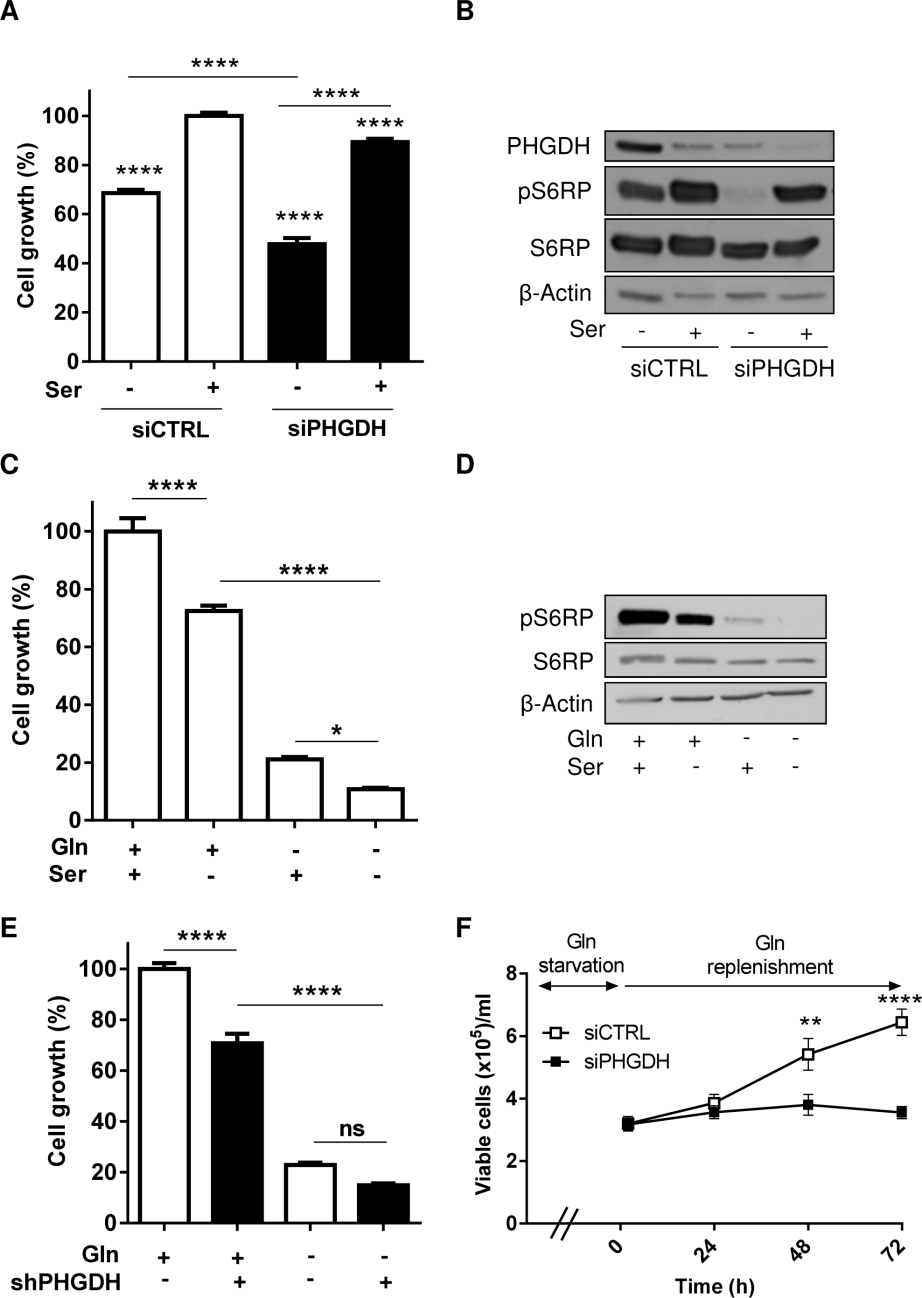
Both exogenous serine and PHGDH expression support leukemia cell growth (**A**) Bar graph represents the growth (%) of HL-60 leukemia cells transfected with either control or PHGDH siRNA and incubated for 48 hours in a medium with or without serine (*n* = 3). (**B**) Representative immunoblots for PHGDH and phospho-S6RP in the corresponding culture conditions. Bar graphs represent the growth (%) of HL-60 leukemia cells (**C**) incubated in a serine-free (or serine-containing) medium (*n* = 3) or (**E**) transfected with a PHGDH shRNA (or a control shRNA), and incubated for 48 hours in a medium with or without Gln (*n* = 2). (**D**) Representative immunoblots of phospho-S6RP in the different conditions described in C; the immunoblot experiments were repeated 2–3 times with similar results. (**F**) Effect of Gln replenishment on HL-60 cell growth after 72 hours Gln withdrawal and transfection with either PHGDH or control siRNA (*n* = 6).

To better understand the reasons of the serine pathway upregulation we investigated the role of endogenous and exogenous serine in leukemia cells. We first examined whether HL-60 leukemia cells were sensitive to a reduction in serine concentration by using serine-deprived medium combined or not with PHGDH-targeting siRNA. We found that serine deprivation inhibited cell growth by 31% and that transfection of PHGDH-targeting siRNA further decreased the extent of cell growth by 20% (Figure 4A). This effect was probably underestimated since serine deprivation itself led to an increase in PHGDH expression, and PHGDH siRNA failed to completely prevent this upregulation (see Supplementary Figure 2E). Still, we found that in HL-60 cells the combination of serine deprivation and PHGDH siRNA completely inhibited the phosphorylation of S6RP used as a marker of mTOR activation (Figure 4B). To further illustrate the critical role of serine in leukemia cells, we examined the combined effects of Gln-starvation and either serine withdrawal or PHGDH siRNA on cell growth. We identified additive growth inhibitory effects of glutamine and serine deprivation (Figure 4C) associated with a complete inhibition of S6RP phosphorylation (Figure 4D). When we combined glutamine deprivation and PHGDH silencing (using dedicated shRNA, see Supplementary Figure 2F), both treatments led to cell growth inhibition (Figure 4E). Glutamine withdrawal alone however led to a dramatic anti-proliferative effect that rendered difficult the detection of an additive cell growth inhibition from PHGDH knockdown (Figure 4E). We thus set up an experiment to instead examine the cell re-growth *after* the episode of Gln withdrawal and PHGDH silencing. In these conditions, we could document that PHGDH silencing prevented the re-initiation of cell growth in the 48–72 hours following re-exposure to Gln (Figure 4F).

### Serine contributes to protect leukemia cells against ROS production

**Figure 5:**
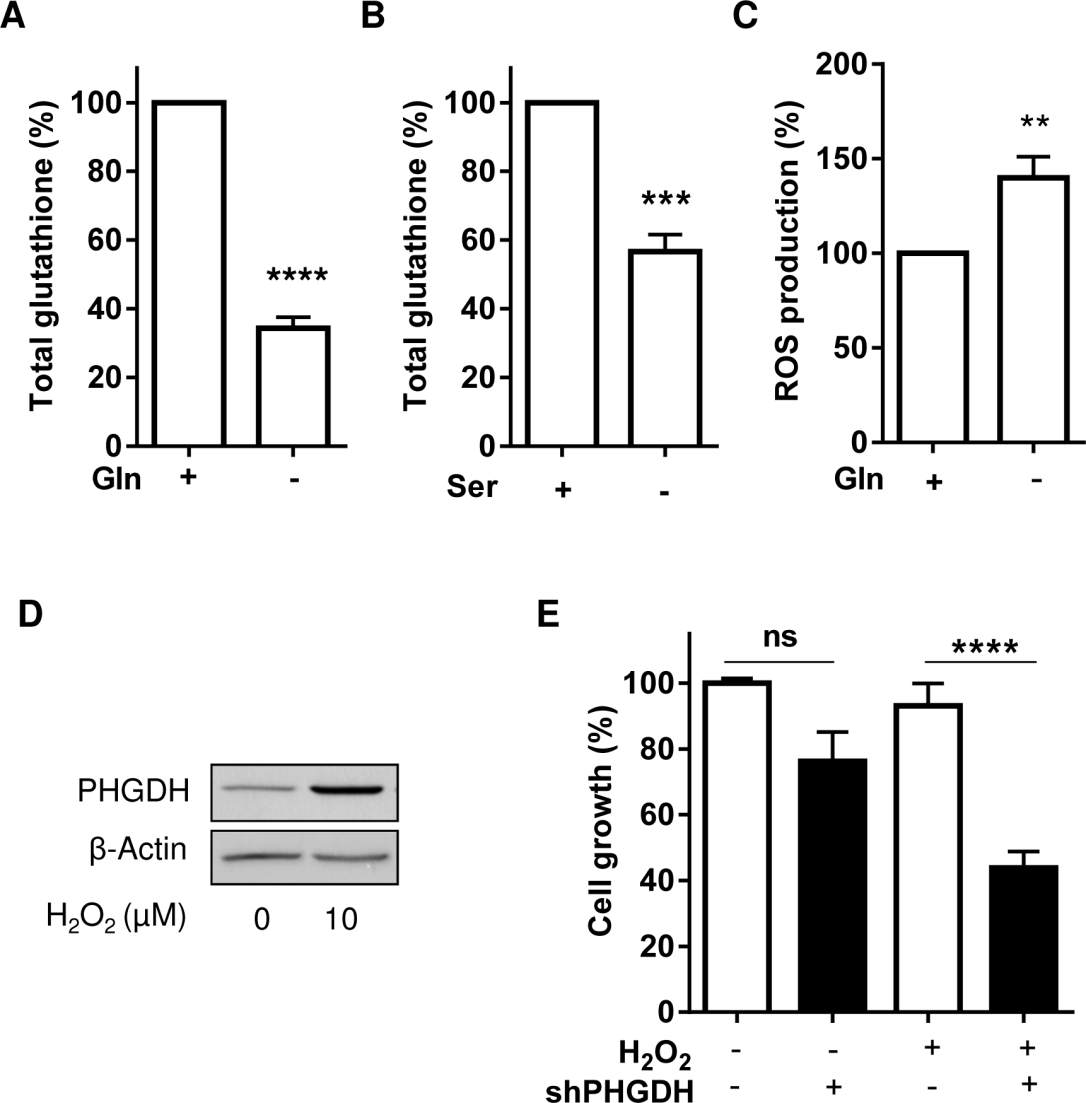
PHGDH expression allows to reduce the extent of oxidative stress induced upon Gln withdrawal Bar graphs represent the amounts of total glutathione in HL-60 leukemia cells exposed to a medium (**A**) with or without Gln (for 6 hours) and (**B**) with or without exogenous serine (for 48 hours) (*n* = 3). (**C**) ROS production (as determined using the H_2_- DCFDA probe) in HL-60 leukemia cells exposed for 6 hours to a medium with or without Gln (*n* = 4). Effects of H_2_O_2_ (10 μM, 48 hours) on (**D**) the expression of PHGDH in HL-60 cells and (**E**) on the growth of HL-60 cells transfected with control or PHGDH shRNA and treated with L-asparaginase.

Among the metabolic intermediates resulting from serine synthesis (and potentially relevant in non-proliferating Gln-deprived leukemia cells), we focused on the glycine/cysteine synthesis (see Figure 3B). Serine is indeed a precursor of these two amino acids that are necessary to form the anti-oxidant entity glutathione (GSH). We actually found that the amount of GSH was sensitive to change in Gln and serine availabilities (Figure 5A and 5B) and that ROS production was increased in Gln-deprived leukemia cells (Figure 5C). We also documented that cell exposure to H_2_O_2_ led to PHGDH protein upregulation (Figure 5D) and further increased the cytotoxic effects of PHGDH silencing in L-ase-treated cells (Figure 5E), thereby proving the role of PHGDH in the antioxidant defenses of leukemia cells.

### Reducing the serine availability increases the anti-proliferative effects of pharmacological inhibitors of the glutamine metabolism both *in vitro* and *in vivo*

**Figure 6:**
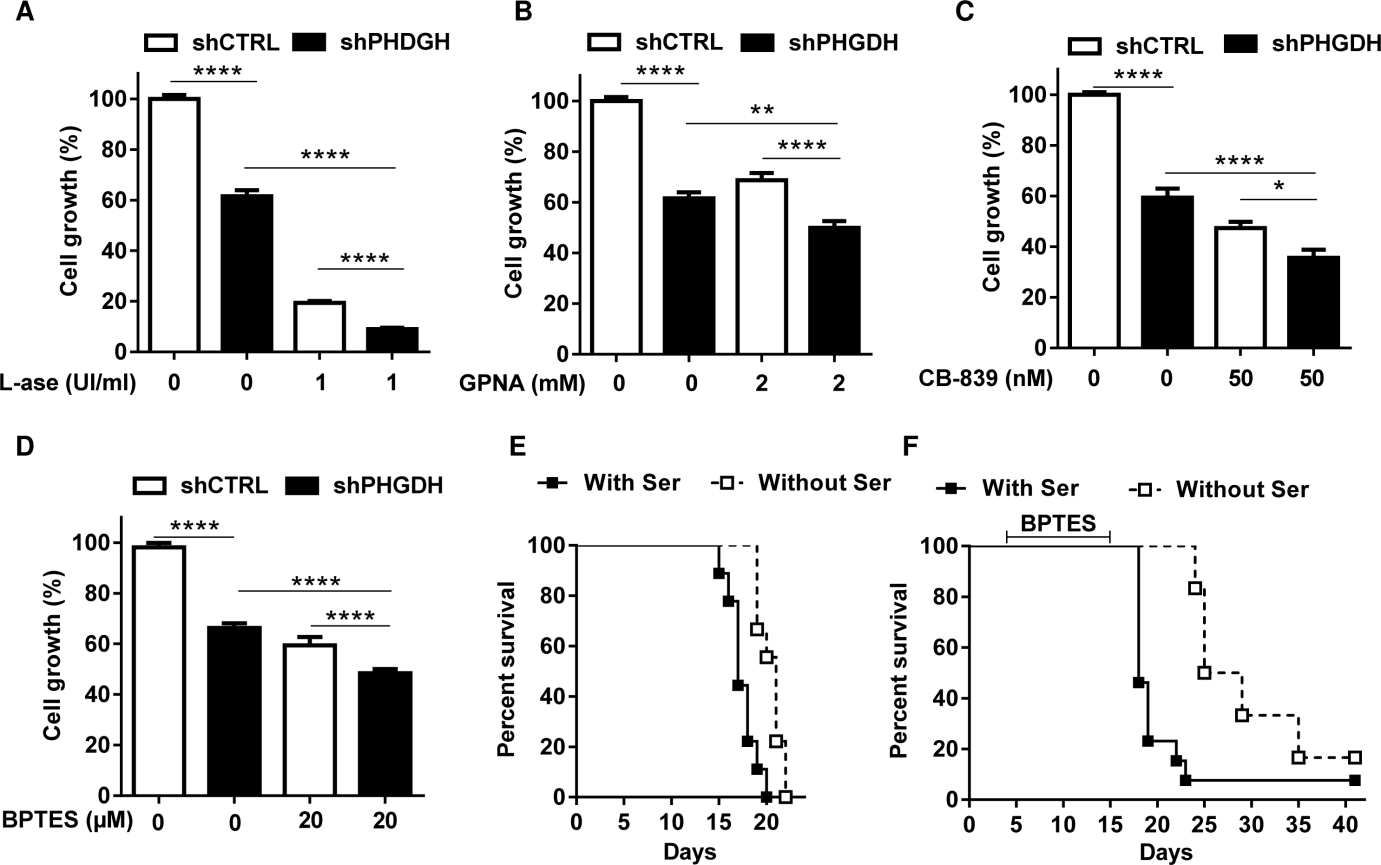
PHGDH silencing and serine deprivation inhibit leukemia cell growth and reinforce the effects of inhibitors of Gln metabolism Bar graphs represent the growth (%) of HL-60 leukemia cell clones expressing either control or PHGDH shRNA and incubated for 48 hours in a medium with or without (**A**) L-asparaginase, (**B**) GPNA, (**C**) CB-839 and (**D**) BPTES (*n* = 3). Kaplan-Meier curves depicting the impact of a serine/glycine-free (*vs.* control) diet on the survival of mice injected with Ba/F3 leukemia cells (**E**) without any other treatment (***P < 0.001, *n* = 9 mice per group) or (**F**) including a daily i.p. administration of 10 mg/kg BPTES (from day 5 to 15) (*P < 0.05, *n* = 6 mice per group).

We finally evaluated the effects of various pharmacological inhibitors of Gln metabolism on the proliferation of PHGDH knock-down leukemia cells. We found that the anti-proliferative effect resulting from PHGDH silencing (−40%, *P* < 0.001) was significantly increased in the presence of either asparaginase or inhibitors of glutamine uptake (GPNA) and glutaminase (CB-839, BPTES) (Figure 6A–6D). We then reasoned that if the serine pathway was so critical to support tumor cell proliferation when the metabolism of Gln was inhibited, serine starvation could have detrimental effects on the *in vivo* tumor growth when combined to the inhibitor of glutaminase BPTES. The poor tumorigenicity of HL-60 leukemia cells *in vivo* led us to consider another leukemia cell line (Ba/F3). We first validated *in vitro* that this other cell line was similarly sensitive to the combination of serine deprivation and BPTES; the effects were actually even more pronounced than those observed in HL-60 cells (see Supplementary Figure 3A–3D). We then evaluated *in vivo* whether serine starvation could prevent the adaptation of mice to the inhibition of the glutamine metabolism. We found that the dietary restriction of serine and glycine prolonged the life of Ba/F3 leukemia cell-bearing mice (Figure 6E). More importantly, when BPTES was administered to these mice, although it only slightly influenced the mouse survival, mice on the diet deficient in serine and glycine rapidly relapsed when the administration of the glutaminase inhibitor was stopped (Figure 6F).

## DISCUSSION

This study identified serine as a critical amino acid for leukemia cell survival and growth, in particular when the metabolic use of Gln is inhibited. We showed indeed that Gln withdrawal or pharmacological inhibition of Gln metabolism exerts profound anti-proliferative effects but limitedly induces leukemia cell death. Together with the maintained activation of the mTOR pathway despite Gln withdrawal, the increase in serine requirement underlines this mode of resistance. This observation is distinct from the paradigm of serine stimulating glycolysis to compensate for the inhibition of oxidative phosphorylation (OXPHOS) in some cancer types [19]. Instead, we showed here that when leukemia cells are deprived of Gln, serine requirements are increased to allow GSH synthesis to clear ROS and ensure cell survival till restoration of Gln availability.

In this study, we identified a reverse correlation between Gln concentration and the expression of two major enzymes of the serine pathway, PHGDH and PSAT in several leukemia cells. Stimulation of the EIF2/ATF4 axis appears as the very likely trigger of the increased need of serine in Gln-deprived leukemia cells. ATF4 that we found to be >10-fold more abundant in the absence of Gln, is a transcription factor known to interact with the PHGDH promoter [20]. Also, the co-detected increase in phospho-EIF2 (known to drive ATF4 expression [20]) is described as a consequence of an increase in uncharged tRNA in cells facing a deficit in amino acids [20, 21], a process supported by our observation of the elevated expression of various aminoacyl tRNA synthetases (see Figure 3A). To understand the role of serine in leukemia cells, we genetically silenced PHGDH and/or used serine-deprived culture medium. We found that the inhibitory effects of PHGDH silencing and serine withdrawal on leukemia cell proliferation were additive, if not synergistic (see Figure 4A–4B), revealing the high dependence of leukemia cells on both endogenous and exogenous serine. Also, when either mode of reducing serine availability was used, it greatly increased the anti-proliferative effects of Gln withdrawal (Figure 4C–4F) and of pharmacological inhibitors of Gln metabolism (Figure 6A–6D). Since cell proliferation was abrogated in the absence of Gln, a role for the upregulated serine pathway in the biosynthesis of nucleotides and metabolic intermediates was unlikely. Instead, we documented the contribution of serine to the cellular antioxidant defenses as a precursor of glycine and cysteine, two constituents of glutathione (GSH) [22, 23]. The role of the serine pathway in leukemia cells thus appears quite different from its recently elicited role in melanoma and breast cancer cells where it represents a major source of α-ketoglutarate to fuel the TCA cycle [19, 24, 25]. It is also noteworthy that in our leukemia cell models, inhibition of the Gln metabolism and the associated upregulation of the serine pathway did not stimulate glucose metabolism contrary to experimental solid tumor models where serine, by binding and activating the pyruvate kinase isoform PKM2, was reported to promote the glycolytic flux [20, 21, 26, 27]. The reasons for this discrepancy are unclear but in leukemia cells, the uncoupling between glycolysis and glutamine metabolism certainly represents an Achilles' heel. This also indirectly underscores that leukemia cells, although often described as a prototypical model of glycolytic cells, requires Gln to proliferate and importantly that Glc can not compensate for a deficit in Gln availability.

Preventing the escape routes to glutamine withdrawal makes sense for the treatment of hematological malignancies and confirms the interest to combine the inhibition of Gln metabolism and of serine availability. Interestingly, since PHGDH inhibitors are not yet available, we fed leukemia-bearing mice with a diet deficient in serine and glycine, and documented that this diet combined with the daily administration of BPTES was sufficient to significantly prolong the life of treated mice. Of note, BPTES was stopped when leukemia-bearing mice fed the normal chow diet started to show signs of morbidity. Whether mice fed with the serine/glycine-deficient diet would live longer if BPTES was maintained, will need to be addressed in further studies. Interestingly, the Ba/F3 leukemia cells that we used in our *in vivo* experiments, expressed the Bcr-Abl tyrosine kinase, a hallmark of CML while most leukemia cell models used *in vitro* are of the AML type indicating that our findings could have a broad applicability in hematological malignancies. Altogether, these data support the concept of oncogene-activated signaling pathways converging towards a similar adaptation of leukemia cell metabolism involving not only glucose but also Gln and serine to support rapid cell division.

In conclusion we provide evidence that inhibition of Gln metabolism exacerbates the leukemia cell dependence for serine. Both intracellular serine synthesis and exogenous serine participate to the pool of serine in leukemia cells, and reduction in either source (ideally in both) exerts potent growth inhibitory effects, in part because of a non-opposed oxidative stress. Altogether, this study underlines that tumor metabolic plasticity also concerns leukemia cells and supports the combined use of drugs targeting Gln metabolism with modalities aiming to reduce serine availability in hematological malignancies. Of note, besides pharmacological PHGDH inhibitors that are currently under development, serine-free or serine-low diet could be envisaged throughout the leukemia treatment; low protein-diets supplemented with drinks containing essential amino acids are already in use for patients suffering of phenylketonuria and could be adapted to reduce exogenous serine availability.

## MATERIALS AND METHODS

### Leukemia cell culture

Different types of human leukemia cell lines were used: promyelocytic leukemia cells (HL-60), acute monocytic leukemia cells (THP-1), myelogenous cells (KG1a), acute monocytic cells (MV4–11) and erythromyeloblastoid leukemia cells (K-562). These leukemia cells were routinely cultured in RPMI Glutamax (Life Technologies) supplemented with 10% FBS. The Ba/F3 cell line transfected with Bcr-Abl (a gift from Dr. K. Bhalla, MCG Cancer Center, Medical College of Georgia, Augusta, GA, USA) was cultured in RPMI1640 medium supplemented with 10% FBS and 1% non-essential amino acids solution (Life Technologies). For the experiments requiring medium adaptation, Dulbecco's Modified Eagle Medium (DMEM) powder (Sigma) was used to generate media containing glucose (10 mM) and/or glutamine (2 mM), and MEM (Sigma) was used to produce medium containing serine or not; dialyzed serum (10%) was used in these experiments.

### Mice

All the experiments involving mice received the approval of the university ethic committee (approval ID 2012/UCL/MD005) and were carried out according to national animal care regulations. Female BALB/c byj were purchased from Janvier. 1 × 10^6^ Ba/F3 cells were injected intravenously in the tail vein. One week before Ba/F3 injection, they received a diet containing no glycine, no serine versus a control diet (Test Diet).

### Drugs

L-Asparaginase (Paronal^®^) was from Takeda and gamma-l-Glutamyl-p-Nitroanilide (GPNA) was from Sigma. CB-839 and BPTES were synthesized in our lab as described elsewhere [28].

### Cell density

Cell growth was measured using PrestoBlue (Invitrogen). Cells were seeded at 1 × 10^5^ cells/ml or 2.5 × 10^5^ cells/ml in 96-well plates. After 48 hours, 10% PrestoBlue was added to each well. After 2 hours at 37°C, fluorescence intensity was measured. Cells were also counted using Cellometer Auto T4 cell counter from Nexcelom wherein viable cells were enumerated by Trypan blue exclusion.

### Flow cytometry

For each condition (Glc/Gln-, Glc- or Gln-containing medium), 1 × 10^6^ of cells were collected and fixed in cold ethanol 70% (in PBS). After 2 hours at −20°C, cells were washed twice in MACS buffer and finally were stained with Ki-67 for 30 min at 4°C. After two washes, cells were incubated with secondary antibody (Alexa 488) for 30 min at 4°C, washed twice and resuspended in 500 μl of MACS buffer. Annexin V/PI labelling was carried out according to manufacturer's protocol (Sigma). BD FACScalibur was used to process all samples (1 × 10^4^ events/sample) and analysis was performed using FlowJo software 7.2.2.

### Western blotting

Western blotting experiments were carried out as reported elsewhere [29]; primary antibodies against the following proteins were used: PHGDH (dilution 1:2000, #HPA021241, Sigma), PSAT (dilution 1:1000, #H00029968-A01, Novus Biologicals) pS6RP (dilution 1:2000, #2211, Cell Signaling), S6RP (1:2000, #2217, Cell Signaling), pEIF2 (dilution 1:1000, #9721, Cell Signaling), ATF4 (dilution 1:1000, #11815, Cell signaling) and β-Actin (dilution 1:10000, #A5441, Sigma).

### 2D-DIGE

HL-60 were incubated either in the absence of glucose or glutamine for one week with renewal of the culture medium every 48 hours. Cells were collected and lysed in with DIGE labelling (DLA) lysis buffer (7 M urea, 2 M thiourea, 4% CHAPS and 30 mM Tris, pH 8.5). Protein labelling with Cy dyes and 2D-electrophoresis were carried out as previously described [30–32]. The 2D-gels were then scanned using a Typhoon FLA 9500 while the images were analysed using the DeCyder software. To identify protein of interest, a min 1.5- fold change in abundance was imposed together with a P < 0.05 (Student's *t*-test). Corresponding spots were then picked and identified by mass spectrometry after trypsin digestion according to a protocol described elsewhere (GIGA Proteomics Facility, ULG, Belgium).

### Gene silencing and cell transfection

Cells transfection was achieved by using Amaxa Nucleofector kit V from Lonza according to the manufacturer's protocol. PHGDH siRNA were from Thermo Scientific Dharmacon (ON-TARGET plus Human PHGDH, smart pool) and PHGDH shRNA was from Origene. Cells transfected with shRNA were selected by puromycin treatment and further diluted to obtain clonal population.

### Metabolic parameters

Glucose, lactate and glutamate concentrations were measured using enzymatic assays (CMA Microdialysis AB) and a CMA 600 analyzer (Aurora Borealis). Total glutathione was measured using a dedicated quantification kit (Enzo). Oxygen consumption rate (OCR) and extracellular acidification rate (ECAR) were performed using the Seahorse XF96 plate reader. Briefly, after 24–48 hours of pre-incubation in the indicated medium, 1.5 × 10^5^ HL-60 cells were seeded in 96-well plates coated with Cell-Tak™ Cell and Tissue Adhesive (Corning). After equilibration in unbuffered DMEM with either Gln or Glc (or both) at 37°C in a CO_2_-free incubator, OCR and ECAR were measured.

### Statistical analysis

Data are expressed as mean ± SEM of at least three independent experiments unless otherwise indicated. Student *t*-test, one-way or two-way ANOVA (Bonferroni's post hoc test) tests and Log-rank (Mantel-Cox) method (for survival curves) were used for statistical analyses; *P < 0.05, **P < 0.01, ***P < 0.001, ****P < 0.0001.

## SUPPLEMENTARY MATERIALS FIGURES


