# Supplementary material for: Reducing the serine availability complements the inhibition of the glutamine metabolism to block leukemia cell growth
Source: Oncotarget. 2015 Nov 28;7(2):1765–76. doi: 10.18632/oncotarget.6426 (PMC4811496; doi:10.18632/oncotarget.6426)
Supplement: Supplementary file 1 [file oncotarget-07-1765-s001.pdf]

Reducing the serine availability complements the inhibition of the glutamine metabolism to block leukemia cell growth

Supplementary Materials

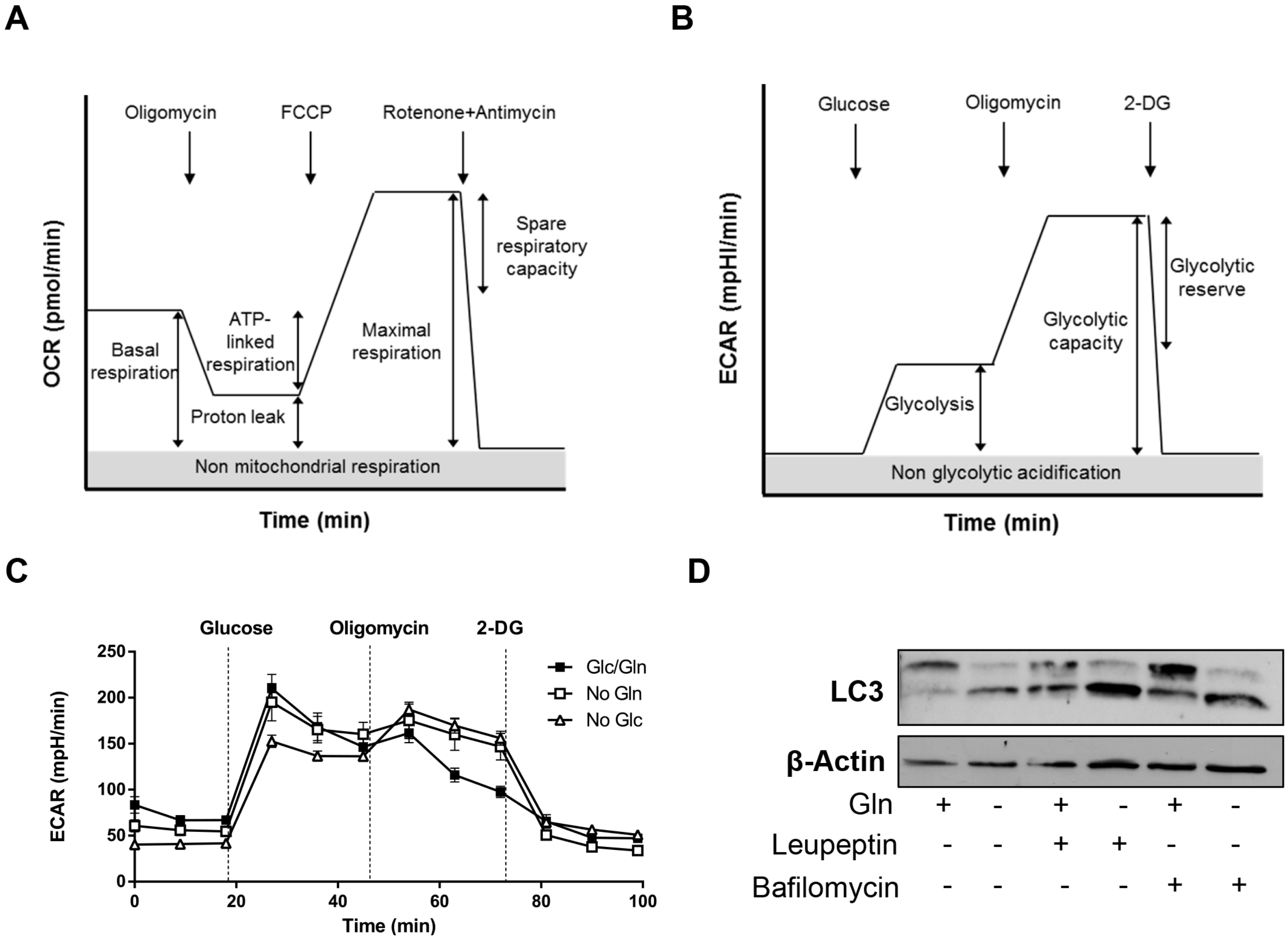

Supplementary Figure S1: Seahorse-based metabolic profiling and autophagy detection in Gln-deprived HL-60 leukemia cells. (**A**) The oxygen consumption rate (OCR) is measured, first without any drugs to determine the basal respiration. The injection of olygomycin, an inhibitor of ATP synthase, helps to calculate the ATP-linked respiration and the proton leak. The FCCP injection reveals the maximal respiration while rotenone (complex I inhibitor) and antimycin (complex III inhibitor) are used to determine the non-mitochondrial O_2 consumption. (**B**) Measurement of the extracellular acidification rate (ECAR) upon glucose injection allows to evaluate glycolysis. The ECAR difference before and after olygomycin injection reflects the glycolytic reserve. Further to 2-DG (a glycolysis inhibitor) injection, the non-glycolytic ECAR can be calculated. (**C**) ECAR measurements of HL-60 pre-cultured in the presence of both Gln and glucose, in the absence of Gln and in the absence of glucose. (**D**) Representative LC3I and LC3II immunoblotting experiments from HL-60 cultured with or without Gln. This experiment was repeated twice with similar results.

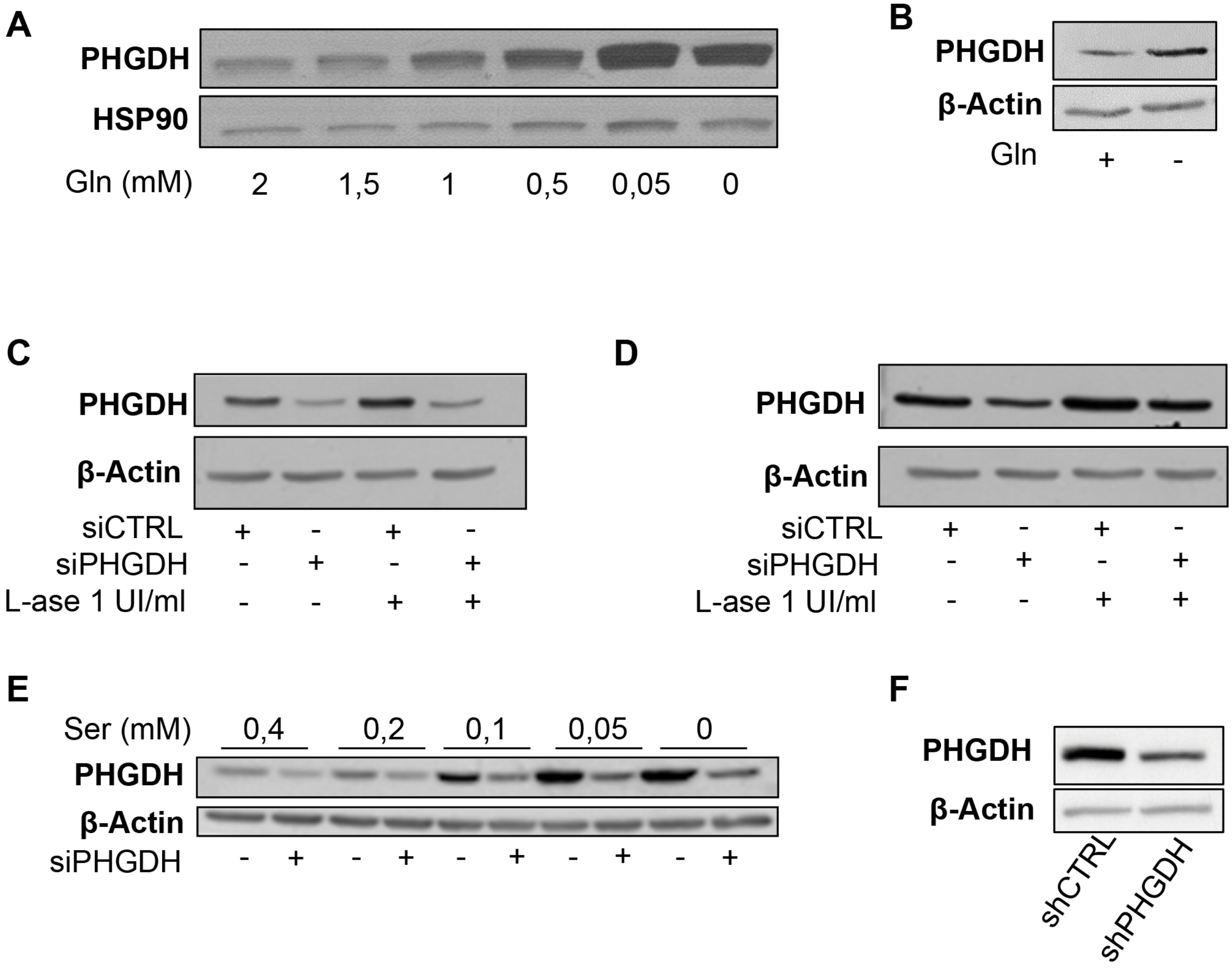

Supplementary Figure S2: PHGDH expression in leukemia cells in response to Gln deprivation or PHGDH silencing. Representative immunoblots (**A**) in THP-1 leukemia cells incubated for 48 hours in medium containing the indicated decreasing concentrations of Gln and (**B**) in K-562 leukemia cells cultured with or without Gln. Representative immunoblots depicting the effects of L-asparaginase (and PHGDH siRNA treatment) in (**C**) KG1a and (**D**) MV4-11 leukemia cells. (**E**) Representative immunoblot depicting the effects of 48h serine deprivation (and PHGDH siRNA treatment) in HL-60 leukemia cells. (**F**) Representative immunoblot depicting the effects of a PHGDH shRNA in HL-60 leukemia cells.

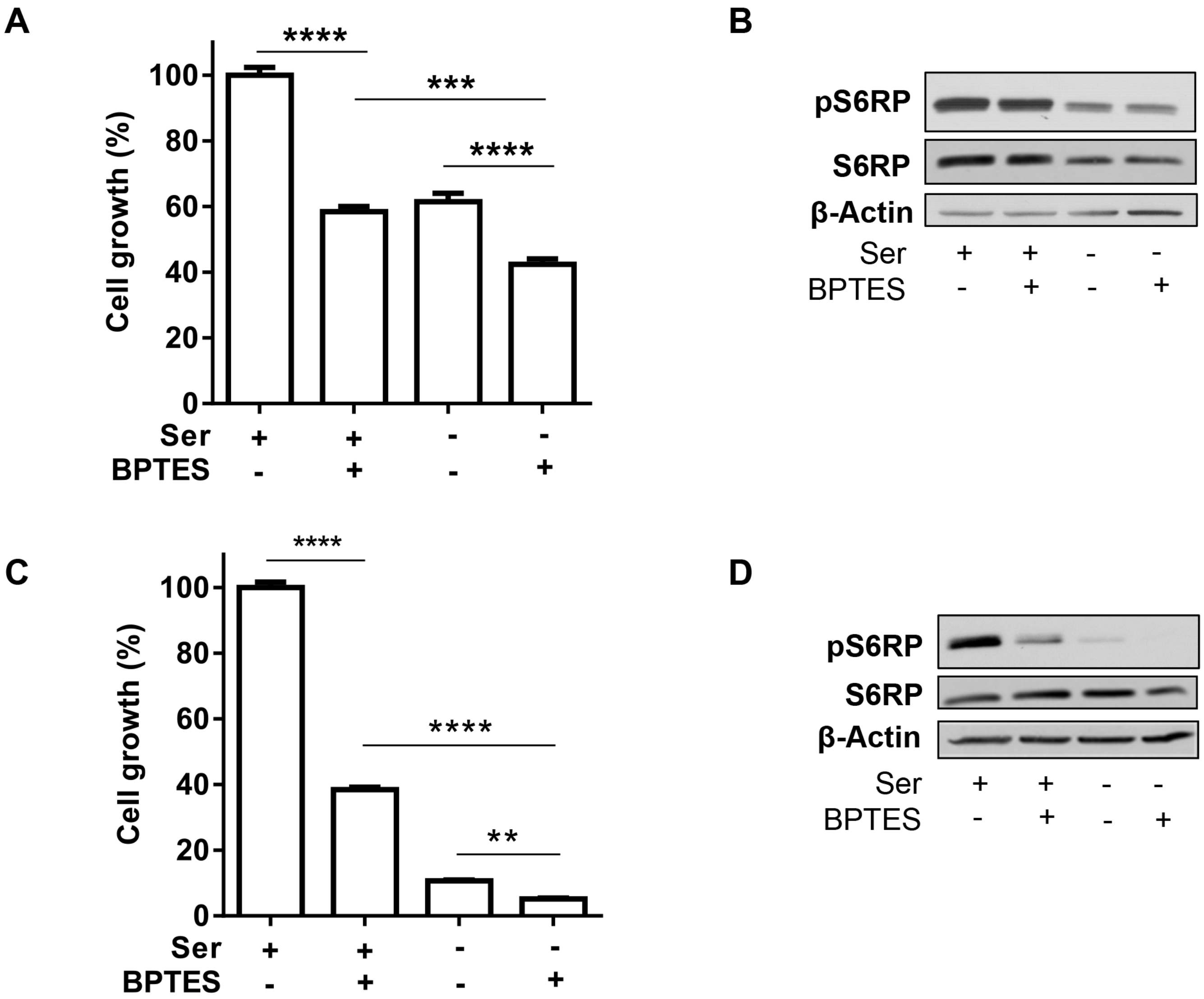

Supplementary Figure S3: Effects of serine deprivation and/or treatment with the glutaminase inhibitor BPTES on HL-60 and Ba/F3 leukemia cells. Bar graphs represent the growth (%) of (**A**) HL-60 and (**C**) Ba/F3 cells after 48 hours of the indicated treatments. Representative immunoblots depicting the extent of phospho- S6RP in corresponding (**B**) HL-60 and (**D**) Ba/F3 cells.
